# “I tried to control my emotions”: Nursing Home Care Workers’ Experiences of Emotional Labor in China

**DOI:** 10.1007/s10823-022-09452-4

**Published:** 2022-02-18

**Authors:** Zhe Yan

**Affiliations:** Department of Contemporary Chinese Studies, University of Würzburg, Am Hubland, 97074 Würzburg, Germany

**Keywords:** China, Long-term care, Direct care workers, Emotional labor, Filial piety/*xiao*, Professionalism, Reciprocity

## Abstract

Despite dramatic expansions in the Chinese nursing home sector in meeting the increasing care needs of a rapidly aging population, direct care work in China remains largely devalued and socially unrecognized. Consequently, scant attention has been given to the caregiving experiences of direct care workers (DCWs) in Chinese nursing homes. In particular, given the relational nature of care work, there is little knowledge as to how Chinese DCWs manage emotions and inner feelings through their emotional labor. This article examines the emotional labor of Chinese DCWs through ethnographic data collected with 20 DCWs in one nursing home located in an urban setting in central China. Data were analyzed using conventional content analysis and constant comparison. Participants’ accounts of sustaining a caring self, preserving professional identity, and hoping for reciprocity revealed implicit meanings about the often-conflicting nature of emotional labor and the nonreciprocal elements of care work under constrained working conditions. Importantly, the moral-cultural notion of *bao* (报 norm of reciprocity) was found to be central among DCWs in navigating strained resources and suggested their agency in meaning-construction. However, their constructed moral buffers may be insufficient if emotional labor continues to be made invisible by care organizations.

## Introduction

From the 1950s to the 1990s, the Chinese government provided little formal long-term care opportunities for its population outside those welfare recipients who comprised the “Three-No Elders”; i.e. older adults with no children, no income, and no relatives (Feng et al., 2020). Hence, nursing homes in China during this period primarily served as social welfare/social relief institutions. These nursing homes, largely in urban areas and very small in number (e.g. only 870 in 1988), were government-owned, funded, and operated (Chen, 1996). Due both to the historical context and the moral culture of filial piety (*xiao*), institutional care was for a long time stigmatized in China. The current development of nursing homes in China arose largely in response to changing demographics and evolving family structures; particularly in relation to unmet care needs as the number of available family caregivers dwindles. As a result, social attitudes have become more accepting of institutional care, and the cultural meaning of subcontracting *xiao* also began to undergo a transformative reinterpretation; including especially a broadened concept of *xiao* which suggests that the high cost of professional institutional care allows institutional care to be viewed increasingly as a privilege and less as a family stigma (Lan, 2002; Zhan et al., 2008).

Situated within these sociocultural contexts, Chinese families now resort to nursing homes for help when older adults are no longer physically functional or cognitively capable. In response to these growing care needs, nursing homes have experienced a great ‘leap forward’ in development in recent years, and concomitant to that, marketization of care services is also on the rise (Alpermann & Zhan, 2019; Shum et al., 2015; Luo & Zhan, 2018). Despite the urgency in meeting the care requirements of China’s rapidly aging population, little is known about how such work is experienced by Chinese DCWs, whose skills and commitments in frontline direct care are among the determinants of the well-being of older adults. In particular, how emotional labor is manifested in DCWs’ daily work given the relational nature of caregiving is inadequately addressed. This article draws on the lens of emotional labor to examine how DCWs manage emotions and inner feelings according to both the expectation of care clients and the requirements of the nursing home in their provision of care.

## Background

### China’s Changing Long-term Care Landscape

Long-term care (LTC) refers to a broad set of paid and unpaid services for persons who need assistance because of a chronic illness or physical or mental disability (Feder et al., 2000). The tradition of *yanger fanglao* (raising a son for elder care in return) is familiar to many Chinese families. This cultural expectation is rooted in the moral ethics of *xiao*. *Xiao* is widely understood and accepted as a foundational element of morality, social life and civil discourse to an extent not always understood outside China (Nie, 2015). Therefore, LTC provisions in China are largely characterized by informal family care mechanisms, supplemented by home- and community-based care, institutional care, and private sources (Gu & Vlosky, 2008). Under the former socialist system there was restricted demand for social welfare services, and older people were expected to seek support primarily from their family and their work units (Wong & Leung, 2012), i.e. employment organizations, which provided ‘cradle to grave’ welfare benefits primarily in the socialist era. However, as China experienced market-oriented transformations in the 1980s and the ensuing socialization of social welfare, LTC was gradually decentralized and diversified to include non-state operators. During this transitional period, nursing homes were no longer dominated by government ownership and started to admit self-funded older adults other than welfare recipients (Feng et al., 2020). Rapid urbanization, increased labor mobility, the one-child policy, and limited capacities of state and local communities have all inflated the demand for formal elder care since the 2000s as families became smaller and dispersed (Wong & Tang, 2006; Feng et al., 2020).

Despite a clear understanding of needs and opportunities, the quality of LTC services is dogged and even undermined by insufficient professional development among the frontline care workforce (Zhang, 2007; Mu, 2012). For instance, in Shanghai’s community LTC services the frontline workforce is composed almost entirely of either local laid-off workers or migrant workers entering the city from the countryside (Wu et al., 2005). Similarly, nursing home DCWs in urban areas are largely laid-off workers and rural migrants (Yan, 2020). The composition of DCWs in China derives from a confluence of two socio-historical contexts. In the process of market reform, many workers in state-owned factories were laid off in the 1990s and were left precarious by a lack of reemployment programs. Due to both a lower threshold for employment and a growing need for care work in urban areas, many of these workers entered the care sector as a last resort. This trend was further bolstered by the lifting of restrictions for rural peasants to migrate internally, with the result that many ended up in care services which urbanites considered an undesirable employment option. As a result, the pool of workers in Chinese nursing homes was found less equipped for care work than their counterparts in Western countries (Song et al., 2014).

### Emotional Labor in Context

Hochschild (1983) describes emotional labor in the service economy as the requirement for workers to orient their inner emotions and outer emotional displays in alignment with a set of occupational requirements. The individual compares and measures experience against often idealized expectations, and the attempts to reduce emotive dissonance are periodic clues to rules of feeling (Hochschild, 1979). This process is regulated by ‘feeling rules’; i.e. a socialized guide to appropriate emotional displays in particular situations and within particular roles and manifested in ‘surface acting’ and ‘deep acting’ (Bailey et al., 2015). Surface acting refers to the superficial production of a socially desirable emotional display, and deep acting refers to the adaptation of inner feelings to align with occupational demands (Hochschild, 1983: 33; Bailey et al., 2015). In other words, feeling rules are guidelines for the assessment of fits and misfits between feeling and situation (Hochschild, 1979).

The concept of emotional labor is widely discussed in the profession of nursing and paid care work, with both positive and negative consequences for the care practices of DCWs (Lopez, 2006; Roitenberg, 2021; Bailey et al., 2015). Due to a lack of time and space, emotional labor becomes ‘invisible and uncodified’ in care work (Gray & Smith, 2009). Further, care work is disdained as dirty work and of low status (Jervis, 2001). Within these constraints, DCWs develop strategies and construct meanings from their experiences of caregiving. In U.S. assisted living facilities, DCWs were found to elevate the status of their jobs by minimizing the importance of low wages and emphasizing the value of care work and the qualities they possessed to perform it (Ball et al., 2009). This is similarly reflected in paid companions’ effort to maintain the ‘caring self’ in Canadian residential long-term care (Funk & Outcalt, 2020). Stacey’s (2005) study on home care workers in the U.S. revealed that they have a conflicted, often contradictory, relationship with their labor; workers in her study identified constraints at work, but they also reported rewards from which they draw dignity. In a private residential care home in England, DCWs considered the emotional labor encouraged by their employer as an extension of their natural loving and caring qualities (Johnson, 2015). Reframing emotional labor as personal qualities (by both workers and their employers) justifies economic devaluations of care work, but also allows DCWs to defend the interests of their care recipients through moral labor (Johnson, 2015). However, organizations do not always coercively require emotional labor. Bureaucratic rules and procedures can also be oriented toward supporting relationships without imposing feeling rules through what Lopez (2006) calls organized emotional care.

Emotional labor intensifies when DCWs, while caring for dying persons and their families, have to juggle conflicting role identities between the caring person and the caring professional. In this context, research suggests that employees struggle to find the space and time to deal with personal grief, because the tensions between emotional care and organizational constraints promote surface expressions rather than authentic emotional care (Funk et al., 2017, 2018). Though DCWs are encouraged by administrators to spend time with residents to forge a family-like atmosphere, it proves to be nonreciprocal as DCWs are discouraged to mourn the death of residents like a family member because grief means a potential disruption to an orderly workflow in the eyes of nursing home managers (Ball et al., 2009; Dodson & Zincavage, 2007).

Through working with their emotions, DCWs also learn to get daunting care tasks done. While some DCWs used depersonalization as a strategy to objectify patients and detach from emotions to complete tasks more quickly and make the work less upsetting in response to the unrewarding and repetitive nature of care work (Lee-Treweek, 1997), others generated authentic emotional attachments to residents despite obstacles and used emotions as rhetorical resources to obtain compliance from residents who are less cooperative and to cast their work and selves in a positive light (Rodriquez, 2014). These interactional processes of emotional labor enable DCWs to reclaim dignity from dirty work. Therefore, the relational aspect of emotional labor highlights that individual working subjects do not operate in isolation, but instead interact with others in their workplace (Roseman, 2019).

There is a dearth of research grounded in the emotional experience of Chinese DCWs. Existing research predominately centers on community-based services and familial care in developed regions, but provides little insight in formal institutional settings. While they unveil the demanding and rewarding aspects of care work (Chen et al., 2018; Hong, 2017; Shea & Zhang, 2016; Chen et al., 2017; Wu et al., 2020), it is not clear how Chinese DCWs experience and manage emotions in navigating heavy workloads and negative social perceptions of care work on the one hand, and the nursing home expectation of compassionate, supportive, and patient workers on the other. To fill this knowledge gap, this article uses a qualitative approach to identify and explicate different conceptual categories in understanding DCWs’ emotional experiences within the context in which familial caregiving is still culturally desirable and the marketization of care services is increasing.

## Methods

### Study Setting

This study took place in a government-sponsored nursing home located in urban central China. The nursing home was first established in 1949 and it now has a capacity of up to 500 care beds and operates its own medical care unit. It was selected due to its leading role in providing professional care service in the region and its formalized management of the care workforce (e.g. investment in professional training). Since most existing research of elder care service provision in China centers on developed regions such as Shanghai, documenting care work experience in a less developed area helps to enrich the understanding of care practices and contributes to policymaking.

In this nursing home, older adults are categorized according to the level of care needs. In line with the standards set up by the Ministry of Civil Affairs, which supervises elder care nationally, there are three care levels: *zili* (referring to those who are capable of self-care); *jiezhu* (referring to those who need assistive devices for activities of daily living); and *jiehu* (referring to those who require nursing care and are dependent on others for essential aspects of daily living). DCWs in this study primarily care for *jiehu* older adults, thus shouldering heavy care workloads. There were close to 70 older adults living on the care floor when the fieldwork of this study was conducted. The day shift is from 7 am to 5:30 pm and the night shift starts from 5:30 pm. One DCW usually cares for eight older adults. However, this ratio varies depending on the turnover of workers. For instance, during the period of Chinese New Year, the ratio could reach as high as 1:10 due to a temporary shortage of workers because many travel home for family reunions.

### Sampling

Participants of this study were purposively sampled. The head of the nursing home was first contacted and the purpose of the study was introduced. After receiving permission from the nursing home, the care manager, who directly supervises the daily work of DCWs, was then asked to help recruit DCWs. Information regarding this study was circulated among the WeChat (a Chinese multi-purpose instant messaging and social media app) group of DCWs and by word of mouth. DCWs who showed interest in the study were invited for in-depth interviews at either the office of the care manager or in a place of their preference (e.g. canteen or activity rooms). The inclusion criteria of DCWs were that they had worked in the nursing home for at least three months and provided direct care (e.g. feeding, toileting, bathing) to older adults on a daily basis. The employment period of three months was used as a criterion to recruit participants because formal working contracts are signed after three months of probation if the DCWs pass performance evaluations and are willing to continue working. Additionally, three months would likely provide participants with a relatively good understanding of the organization and its procedures for nursing home care work. DCWs who had not worked up to three months or had not completed probation were excluded from the study. At the beginning of each interview, participants were given detailed information of the study and their enquiries of the research (if any) were addressed. A fuller description of sample characteristics can be found in Table 1.

**Table 1.** Demographic characteristics of participants (*N*=20)

Demographic Characteristics	*N (%)*
Age (year)	
20-40	1 (5.0)
40-60	19 (95.0)
Average	47.6
Gender	
Female	18 (90.0)
Male	2 (10.0)
Education Level	
Illiterate	1 (5.0)
Elementary school	3 (15.0)
Middle school	8 (40.0)
High school degree	6 (30.0)
College degree	2 (10.0)
Work experience (years)	
1-5	14 (70.0)
5-10	3 (15.0)
≥ 10	3 (15.0)

### Data Collection

Data of this study were collected through in-depth interviews and participant observations. The fieldwork lasted for four months in total, ranging from the end of 2017 to the end of 2019, and was completed before the COVID-19 pandemic. Through the sampling strategy described above, 20 DCWs were recruited and agreed to be interviewed and participate in the study. Seven DCWs were interviewed more than once throughout the fieldwork. Interviews lasted from half an hour to 2.5 hours in length. Though there was an interview guide, DCWs were encouraged to freely discuss issues that they consider important to their caregiving experiences. Major aspects of the interviews with DCWs included: daily routine of care work, motivation to work in elder care, emotionally rewarding and challenging experiences of caregiving, and their responses in these situations. Questions were informed by the theoretical concept of emotional labor and existing literature on care work. For instance, the aspect of motivation for providing care included questions such as “How did you become a DCW?” and “What are the reasons why you decided to work in nursing home care?” To delve into the emotional aspect of their caregiving experiences, questions such as “How would you describe your relationship with older adults?” “What are the most emotionally challenging and rewarding parts of your work and how do you cope with these challenges?” were asked to encourage participants to share their experiences of emotional labor. After receiving informed verbal consent from participants, interviews were recorded and transcribed verbatim. In the case of those who declined to be recorded, interview notes were taken in their presence. In order to contextualize and supplement the experience of DCWs, informal interviews were also conducted with institutional heads and care managers who supervise DCWs, older adults, and visiting family members. These were mostly conducted casually and recorded in field notes.

Data were also collected through participant observations. On a typical working day, the researcher stayed at the nursing home from 6 am until 10:30 pm. This enabled observation of both day and night shift workers. Throughout the day, observation included participating in the care meeting held in the care manager’s office in the morning and the group checkup of older adults afterwards. The researcher also assisted DCWs in dressing and bathing older adults, and observed worker training sessions. Particular attention was given to the period of the day when DCWs experienced the heaviest workload (e.g. morning hours of getting older adults ready for breakfast) as these moments were likely to reveal authentic interaction between DCWs and older adults. Participant observations were recorded in field notes and were later used for triangulation of the interview data. This research received ethical approval from the mentoring committee of the University of Würzburg.

## Data Analysis

Conventional content analysis was used to analyze collected data (Hsieh & Shannon, 2005). This analytical approach is best suited for studies describing a phenomenon with limited previous literature and orients researchers to the importance of context and an individual’s experience (Hickman et al., 2020; Given, 2008). Specifically, the approach integrates a ‘top-down’ theoretical perspective with a ‘bottom-up’ data-driven approach (Braun & Clark, 2006). The interview transcripts were first read several times to facilitate a holistic understanding of what is said about caregiving experiences. In a second step, codes were derived from segmenting original transcripts into different meaning clusters. These clusters were then rearranged at a more conceptual level into categories. This conceptualization process was realized through creating memos and referencing literature on caregiving with a conscious focus on the context through which the codes and categories were derived. In particular, gerunds were used in facilitating the emergence of processes from DCWs in describing their work. For example, instances of the code ‘emphasizing caring attitude’ were clustered into the theme of ‘sustaining a caring self’ in using constant comparison across interviews. In another example, codes like ‘increasing care cost’ and ‘unwillingness to burden children’ were first grouped into ‘concern over future care’ and later identified as a contributing factor for the theme ‘hoping for reciprocity’.

The rigor of findings in qualitative research is critical. To ensure the rigor of findings for this study, the researcher wrote a manuscript report summarizing the interview analyses and observations in Chinese and shared it within the WeChat group of DCWs to receive feedback on whether it authentically reflected their accounts. The report was also communicated to one nursing home head and one care manager for further refining. Additionally, part of the collected data was shared with colleagues to discuss the process of data analysis. Results were also presented and discussed at a local university.

## Results

Three overarching and interlocking themes were identified: sustaining a caring self, preserving professional identity, and hoping for reciprocity. These themes suggest the implicit meaning and often-conflicting nature of nursing home care work and are described in detail below with narratives from representative DCWs summarized in each theme. Although the data presentation focuses primarily on three DCWs, data analysis is informed by knowledge gained from interviews with all participants and field observations.

### Sustaining a Caring Self

A caring personality is how many DCWs explained their decision to work in elder care. They understood being caring as a necessary precondition for the profession. This section explores how feelings of caring and attachment to older adults orient DCWs toward prioritizing the well-being of older adults by focusing on the caregiving experience of Yu^1^. Yu is 49 and has been working as a DCW for three years. Born in the countryside, like many rural-to-urban migrant workers she moved to the city with her sister in search of better living conditions. They started a street vendor business in the early 1990s, when market reform in China was in full swing. Through arranged dating, Yu married a local man and settled down. The marriage provided her a sense of security as she did not have a local *hukou* (household registration), an institution which categorizes the Chinese population into rural and non-rural registrants and bestows differential welfare benefits based on those registrations. Yu stayed at home to care for her son until he went to middle school, the focus of hope for many one-child families (Fong, 2004). As she had no formal working experience, she took a job as a clerk in a supermarket. One primary motive for her to work in a nursing home was to mitigate her regret for not being able to provide end-of-life care for her father due to her own family obligations.

I did not fulfill *xiao* for my own father. This makes me feel guilty. In our village people gossiped about my absence. Taking care of your aging parents is still the norm and working here provides the opportunity to compensate for my personal regrets. I’m genuinely happy to care for the residents here and I consider them as my own parents. You won’t feel tired caring for your own parents. If you only work here for the paycheck, you won’t last long. In fact, many of the DCWs who came at the same time as me have left.

Yu emphasizes the importance of compassion and a caring attitude in providing effective care. However, her emotional goals are often challenged by the relentless care tasks and sometimes untenable demands of older adults. Additionally, she is bothered by the negative social portrayal of care work as dirty work and the disrespect she experiences from older adults and their family members. Despite the reservations by her family members and friends, Yu chooses to remain in elder care, and she finds continued determination through empathetic reasoning.

People like us were referred to as *xiaren* (literally, a low person, i.e. servants) in the old days and I know many still treat us as such. It is the disrespectful attitudes which could kill you! Though we sometimes get mistreated, I have to adjust my feelings and concentrate on helping older adults who are pitiable (*kelian*). They have chronic illness and their family members are away. I am fulfilling their adult children’s filial obligations, but I feel bad when I have to rush from one resident to the next. Will someone do the same (for us) when we get old? Most of us have only one child, and it will conceivably be more difficult and expensive (to get care) in the future.

Though Yu maintains a caring attitude in fulfilling other adult children’s filial obligations, she is consciously aware of and concerned about the unpredictability of her own future care. In response to being treated by older adults and family members as personal servants with little respect, DCWs exercise restraint on their emotions to avoid direct conflict and they are exhorted by the nursing home to do so as they hold a commercial relationship with care clients. However, despite their personal efforts to satisfy such commercial purposes and to follow working protocols which prioritize the interest of older adults over that of their own, the emotional labor needed by DCWs to do so is often overlooked and unrewarded by administrators because it cannot be objectively measured. Moreover, heavy workloads and understaffing further compromise DCWs’ ability to attend to the personal needs of older adults, causing moral distress in managing emotions.

Care manager Chang, who directly supervises the work of DCWs, unreflectively imposes additional emotional requirements on DCWs. She assigns DCWs to be ‘in charge’ of older adults in designated rooms, and they are required to directly communicate with family members about daily care practices, and to address concerns from family members. Chang considers this an efficient way to avoid potential escalations of conflicts between family members and the nursing home when dissatisfaction and misunderstandings occur. Since DCWs provide direct care for particular older adults, Chang argues that they are best qualified to interact with family members and they should properly manage their emotions when communicating with clients. This often involves coping with unreasonable complaints and untenable care demands from family members. When asked if she thinks this situation creates extra work for DCWs, Chang indicated that if DCWs were only to handle instrumental care (bathing, feeding, toileting), then they would be underperforming and unprofessional. This view indirectly normalizes emotional labor and mechanically reinforces a caring attitude among DCWs through the mask of professionalism.

Perhaps the most challenging aspect of emotional labor is to manage grief when older adults pass away. DCWs often suppress their felt emotions at work in order to maintain a boundary between personal emotions and professional practice. For Yu, this remains a challenge. When Grandpa Zhu passed away Yu burst into tears because she had been caring for him for the previous three years. However, emotional grief must be managed personally as the routines of care still need to proceed, and so Yu did all she could to refrain from overt emotional expression.

I guess it was because I devoted a lot of emotion into my work. Maybe I *shouldn’t*…I was very uncomfortable after learning of his death and I couldn’t help crying when I talked about him. I tried to control my emotions since I didn’t want to disturb other residents and my colleagues. My feelings for him should be managed personally. Care work is teamwork, and I had to adjust my emotions.

In this situation, Yu chose to suppress her grief to prevent potential spillover effects for both colleagues and other older adults. By suggesting that she *shouldn’t* be too emotionally attached to older adults, Yu was aware of the feeling rule in this context which expected her to control her emotions in favor of the smooth flow of care work. She thus considers emotions as personal, even if they are work-related, and that boundaries should be maintained. The conflicting nature between emotional and professional caregiving undermines the well-being of DCWs without guided channels for emotion management (e.g. coping with grief). Interestingly, the nursing home did later develop a program to help DCWs relieve stress and manage emotions, but Yu and her co-workers perceived that their employer didn’t really care about DCWs as the program overlooked some of their other real needs.

It’s absolutely true that we need to find ways to navigate feelings, otherwise we won’t be able to continue such emotionally draining work. The nursing home has gathered us for activities to help us relax. But these activities are conducted during our lunch break. It is undertaken in the name of supporting us, but we don’t want to attend. We are simply too tired. We just want to sit down and have a real rest!

It becomes clear from the experiences of Yu that a caring attitude and personality could be a double-edged sword. It simultaneously motivates DCWs to provide attentive care and enables the nursing home to normalize such emotional commitment without recognizing the time and effort workers have to invest, and usually without compensation. The next section will show that DCWs sometimes evoke professionalism to deemphasize the emotional nature of care work without losing sight of the well-being of older adults.

### Preserving Professional Identity

Fang is 43 and joined the nursing home after graduating with a nursing degree from a civil affairs vocational school in 1996, just at the time when automatic job assignments (*fenpei*) for qualified graduates in China was ended. She was recommended by her teacher to the nursing home, and has been a DCW there for more than 20 years. She identifies herself as a professional person and considers the nursing home her second home.

I came here right after school. Though I learned all the theories at school, I encountered many difficulties when I started my internship here. It took a while to get used to caring for older adults. The reason why I remain here has a lot to do with the kindness I received from the two heads of the nursing home. I need to stay here and support them. I have my career in this nursing home and I can say I’m good at what I do. Not every person can be a qualified care worker.

In contrast to Yu, Fang’s professional recognition of care work leads her to understand caregiving more in line with a sense of responsibility for the mission of the nursing home than with her emotional attachment to older adults. It is worth noting that this does not mean that Fang is less caring, but rather that her education in nursing orients her to professionally manage emotions under challenging work conditions. Though Fang aligns her inner emotions with the feeling rules of the nursing home (e.g. caring, supportive, patient), she exerts agency by ‘professionalizing’ her inner feelings. For example, Fang made it clear that it is not the older adults, but the nursing home to which she feels stronger emotional attachment.

I think it’s more my attachment to the nursing home than the residents that made me stay. The residents come and go. I try my best to provide good care. Nursing homes cannot control how I feel. The administrators were saying that they don’t understand why we’re still so busy even after they outsourced the cleaning tasks which we had to do before. They assume we would be less busy. But we use the time for meticulous personal care, otherwise family members won’t be satisfied with our nursing home. If no residents stay with us, what would happen?

Though implicit, Fang’s professional orientation is closely tied to market principles which value competition based on service quality. That is why she is concerned with a potential decline of clients. But in her opinion the nursing home cannot engineer emotional care from DCWs because emotionally authentic caregiving cannot be mechanically manufactured by working protocols, but instead takes time and effort to develop. In Fang’s case, professional identity prompts her to provide personal care and also allows her to emotionally detach from older adults when needed. Throughout the fieldwork, she appeared to be more rational in her explanations than emotional when describing her experiences with older adults, including those who had passed away. Commenting on the aspect of care work which involves the decline and the death of older adults, Fang believes this has little negative/emotional impact on her, unlike some other DCWs who express a more caring attitude.

Fang: The decline and death of older adults doesn’t really disturb me emotionally. It actually makes me realize that every person will get old and you cannot imagine what you will be like. What I learned is that you cannot control the complexities of aging. I accept this because of my experience in elder care. For myself, I don’t know when I will get sick and won’t be able to work again. When older adults die, their family members cannot always be there for the final moment. With my work, I can at least contribute to a good death for them.

Interviewer: Is it because you get used to it (the death of older adults)?

Fang: I don’t think I’m insensitive to their conditions. The older adults are with us for so long and we know so much about them. We can easily detect any small changes in them and immediately inform the nurses and doctors. We make sure medical staff are in place to resolve the problems. We also inform their family members and are responsible for a whole series of things.

In addition to caring for older adults by adhering to and internalizing principles of professionalism, Fang also mentioned her relationship with other DCWs and her emotional labor with them. As the head DCW among them, she tries to bolster their willingness to work at the nursing home due to concerns over potential turnover and the inconveniences this causes for the smooth operation of daily care work.

We had several DCWs who left because of serious physical conditions. Yet we still work here as long as we can because we really enjoy working together. We help each other and it makes us feel less tired emotionally. We’re understaffed, and if one person asks for leave then the rest would share the workload. I’m worried that some DCWs might quit. To ensure they stay longer, I don’t directly confront them even when they don’t perform tasks well. I engage them indirectly because it might hurt their feelings if I’m too harsh. I cannot afford to lose them!

At first glance, stabilizing the care workforce should be the responsibility of the nursing home, not individual workers. But their strong sense of professionalism compels DCWs like Fang to exert a sense of autonomy into their labor. To ensure the employment of DCWs, Fang voluntarily takes responsibility to stabilize the workforce even though it is not part of her job description. To avoid hurting her co-workers’ feelings, Fang restrains her own emotions and adjusts the way she interacts with them. The constant worry she has over the state of the care workforce puts extra pressure on her. Some of her co-workers no longer need to work in the nursing home because they have already reached retirement age. Therefore, Fang’s inner emotions are linked not only to feeling and display rules set by the nursing home, but are also sensitive to her recognition of her professional role and the responsibilities that come with it.

We have so far seen the overarching prominence of being caring and professional in the performance of nursing home care work. As a result of the rapid expansion of the institutional care sector in China, both policy requirements and public expectation for the professionalization of the care workforce have positively affected DCWs’ identity formation and meaning construction in the process of providing care. However, their employment in a government-sponsored nursing home has made these workers aware that they themselves will not be similarly cared for by the state due to its retreat in welfare provision and the marketization of care services. Against this background, the next section describes how the notion of *bao* (norm of reciprocity) is used as a coping strategy by DCWs to sustain their committed care for older adults.

### Hoping for Reciprocity

Like many of her generation born in late 1960s, Hong worked in a state-owned factory and thought her life would never deviate from the ‘iron rice bowl’ (lifelong employment guaranteed to state workers in the socialist era before market reforms). But change did come when market reform swept through China in the 1990s and subsequently made many factory workers redundant. Many workers had limited skills and no age advantage for reemployment. The factory Hong worked for encouraged workers to try their luck in the open market (*xiahai*). ‘I took the opportunity, and wanted a different life.’ Hong started a tobacco shop with several friends, and the business expanded rapidly. However, it didn’t last long due to her mismanagement. Hong started as a DCW in the early 2000s as it had few entry requirements.

Hong is 52 and has experience working at different types of nursing homes, including both private and public facilities. She has been a DCW for over 10 years. When first interviewed, Hong kept emphasizing the importance of personal compassion in providing care and how a sense of compassion was reinforced by the training program provided by the nursing home. She seems to be proud of her resilience in managing challenging work conditions and still providing good care, especially in the absence of adult children. Indeed, through the nursing home’s purposeful cultivation of a caring attitude, DCWs have learned to internalize feeling rules and to display their emotions in ways expected by care recipients and required by the nursing home. However, the promotion of family-like bonds between DCWs and older adults is likely to impose extra emotional burdens on DCWs.

One resident I cared for has two sons and they are unfilial and unreliable. What they really want is her money. How unfilial they are! She sold her apartment and moved into the nursing home. I would sometimes cook at home and bring her food. She always told others that I am her daughter. Sometimes I ask myself: can I still count on my child for anything when I’m old? I don’t want to think about it. I only hope I will receive good *bao*.

Similar to other DCWs, Hong is equally concerned about the availability of future DCWs. Due to their daily interaction with aging bodies both physically and emotionally, the thought that ‘their today is our tomorrow’ motivates DCWs to engage in emotional labor to provide authentic care in hope that they themselves would receive good care in the future. The sense of *bao* is strongly felt among DCWs when describing the meaning of their work. It also helps DCWs to navigate their many concerns, e.g. their perceived decline both of *xiao* and of the limited resources for care currently available to them even now. Many believe that their endurance in care work will result in good health and fortune for themselves and could also benefit their loved ones. Such karmic ideas among DCWs help them to navigate daunting care demands and mitigate their experiences of social disrespect for their care work.

However, despite the comfort provided by invoking the moral-cultural notion of *bao*, DCWs are well aware of factors which undermine their future care provision. Both overwhelming structural constraints in the provision of person-centered care, and the continued discounting by institutions of emotional labor, cast shadows on the sustainability and effectiveness of LTC in the eyes of DCWs. Additionally, in contrast to most of the older adults whom DCWs care for (aged between 80 and 90) who have more than one child and who have better pension plans since they were less affected by the market reform, DCWs’ future care is made precarious both by less available family caregivers and inadequate pension benefits. For example, 17 DCWs in this study have only one child, and all but one DCW reported financial concern over their future care arrangements. The social changes wrought by market reform and population policies in China have taken a heavy toll on the current generation of DCWs in ensuring care resources for themselves.

In spite of these concerns, Hong considers both her rich experience and her motivation to update her knowledge in geriatrics as potentially having market value in China’s burgeoning care industry. She even speaks of entrepreneurial opportunities, “The care industry in China is promising, and care work is more formalized. Many family members told us that they wouldn’t know what to do without us. After having enough experience, I want to open my own nursing home one day!” In this hope, she has actively engaged in the cultivation of new DCWs; taking pride in persuading her younger sister, who used to be a businesswoman, to become a DCW, and encouraging her son to consider working in her future nursing home. Her dream of becoming a nursing home operator helps Hong to ameliorate concerns over her own prospects for her future care. Meanwhile, she states that she is sowing the seeds of kindness, and believes in the karmic principle of *haoren you haobao* (a good person will be reciprocated/blessed).

The hope for future reciprocity is difficult to sustain in caregiving when immediate reciprocity cannot be realized. This becomes apparent in conflicts between DCWs’ own familial care obligations and the heavy workloads assigned by the nursing home. For example, Hong’s experience of her husband’s stroke made her emotionally and physically exhausted, reinforcing her earlier experiences of helplessness and unfairness from being laid-off. Due to chronic understaffing at the nursing home, Hong was ‘reminded’ by care managers to professionally balance her role as a caring wife and a caring worker without receiving any substantial support from her workplace.

My husband’s stroke came as a shock to me. I wanted to spend more time to care for him but the care manager told me we’re understaffed and they wouldn’t know what to do if I were absent. I had to rush to the hospital right after work. I couldn’t let older adults under my care feel that something was wrong with me. I had to smile at them, though what I really wanted to do was cry! It might sound silly but I had no time to cry.

Although belief in reciprocity was strongly evoked by DCWs to assuage their concerns over future inadequacies of care resources, this aspect of emotional labor is largely premised on the subordination of DCWs in ensuring the uninterrupted daily operations of the nursing home. As the experience of Hong shows, her personal care needs (e.g. caring for her husband) ironically became secondary in the process of sustaining a caring and professional self in the workplace.

## Discussion

This study explores the caregiving experiences of Chinese DCWs with a focus on how they manage emotions and inner feelings to maintain self-worth and navigate concerns. The emotional labor in care work (e.g. suppressing grief and putting on a smile) is manifested in DCWs’ construction of caring and professional identities. Participant accounts suggest that paid care work is taxing and exerts emotional care burdens on DCWs. While the study findings in part resonate with literature on nursing home care work in other countries, what distinguishes this current study from existing literature is its unique cultural and social context. For one thing, the expectation for family participation (e.g. filial piety) in elder care and the belief in *bao* (e.g. that current commitment in care would be reciprocated) have shaped the emotional landscape of paid care work in China within which DCWs navigate on a daily basis.

The first theme of sustaining a caring self uncovers the deep acting (Hochschild, 1983) of DCWs through both internal construction of fictive-kin ideology and external exhortation from the nursing home. A caring self is also emphasized by paid companions in Canadian care facilities who said they have to love people and cannot just do it because it’s a job (Funk & Outcalt, 2020). This resonates with Yu’s opinion that DCWs won’t work long if only for a paycheck. Though a caring self is found among both institutional and home care workers (Stacey, 2005; Brown & Korczynski, 2017; Johnson, 2015; Ball et al., 2009; Yan, 2020), the emotional labor it takes to both downplay the employment aspects of their role and simultaneously forge caring identities reinforces an unspoken assumption that payment for care is worth less than altruistic forms of care (Funk & Outcalt, 2020); contributing to DCWs’ trapped image of ‘prisoner of love’ (England, 2005). Nonetheless, sustaining a caring self provides meaning to DCWs through both the satisfaction inherent in relationships and through the effect of relationships on care outcomes (Ball et al., 2009), such as ensuring a good death spoken of by Fang. However, different from altruism or voluntarism as motivations for care among DCWs in the West (Dodson & Zincavage, 2007; Johnson, 2015), Chinese DCWs in this study both consciously align emotional labor with the moral ethic of *xiao* to manage inner feelings, and also place themselves as defenders of *xiao*, thus transcending their stigmatized role as simply ‘dirty workers’ (Jervis, 2001). This becomes clear in the moral comfort Yu has found in fulfilling filial obligations to older adults in compensation for her ‘unfilial’ absence in her father’s care. It can be argued that Chinese DCWs tend to be less tolerant of absent families than their U.S. counterparts who were more forgiving of absentee families due to the culture of individualism (Jervis, 2006). But a moral sensibility toward emotional labor is found across cultures, which transforms low status or even ‘degraded’ work into an emotional labor that is viewed by DCWs as challenging, meaningful, and significant for society (Black, 2004).

The second theme of preserving professional identity highlights the complexity of caregiving and problematizes normalization of emotional labor which obscures structural conditions underlying caregiving dilemmas. Tensions between professional ideals and caring identities arise when DCWs are expected to exert detached self-control over their emotions through deep acting (Hochschild, 1983), such as grieving deaths or managing negative internal feelings which arise from ongoing interactions with aggressive older adults. One potential problem this creates is that the normative power of professional detachment stigmatizes those who express grief, and generates ambivalence among workers who are trying to maintain a caring sense of themselves (Funk et al., 2017). It is worth noting that the productive work of detachment (Hochschild, 1983) is not without merit, but needs to be evaluated in a context-sensitive manner. For example, Bailey et al.’ (2015) work on the emotional labor of health-care assistants in dementia care presents detachment as an active emotional choice to put aside certain feelings in the interests of completing one’s job. Indeed, as suggested by Fang, she is not insensitive to older adults but tries to avoid emotional exhaustion by channeling her inner feelings into providing professional care and maintaining the willingness of her co-workers to continue in the work. Moreover, Fang’s perception of holding total responsibility for older adults under her care empowers her to find meaning in care work and counteracts DCWs’ powerlessness in other aspects of their lives (Shenk, 2009). Compared with their counterparts in community-based care programs, DCWs in this study similarly experienced an ambiguous sense of professional identity due to unclear job descriptions (Chen et al., 2017), causing confusion about their professional role. Therefore, the emotional labor it takes to balance caring and professional identities needs to be duly acknowledged and supported by care organizations in order to address polarizations of the caring and professional components of direct care work.

The third theme of hoping for reciprocity relates to nonreciprocal elements of care work such as the subordination of DCWs’ long-term personal interests. Such nonreciprocal experiences of caregiving are found among DCWs in many countries (Dodson & Zincavage, 2007; Banerjee et al., 2015; Jervis, 2006). Though the one-way nature of DCWs-residents relationships posits resident vulnerability as a legitimate basis for DCWs managing their emotional displays, these one-way rules of moral conduct make DCWs vulnerable when residents mistreat them (Johnson, 2015). In other words, emotional labor is encouraged when employers see the potential benefit (e.g. forging harmonious and caring relationships), but discouraged when it threatens care efficiency. This resonates with the process of ‘rationalized aging’ described by Glaser (2019) in which cost-cutting imperatives cleave physical needs from the emotional and relational needs of older adults. Despite market principles in organizing care work, this study adds to the moral discourse of care work from a Chinese cultural perspective. Chinese DCWs evoke the moral-cultural notion of *bao* as an interactional ‘toolkit’ (Swidler, 1986) to appeal to traditional reciprocities in caregiving in mitigation of market pressures imposed on their identity. The hope for reciprocity among Chinese DCWs serves a conciliatory purpose in mitigating the often-conflicting nature between both caring and professional identities and engagement and detachment in paid care work, particularly in emotional labor. Even beyond the immediate context of nursing home dynamics, dramatic social changes in China have also contributed to the vulnerability of DCWs. Many DCWs lost their former lifelong state employment and are now faced with inequalities in obtaining insurance and social benefits that result from being rural *hukou* holders living in urbanized settings; constraining their long-term well-being on an even wider scale (Hong, 2017).

The sense of reciprocity exemplified in *bao* is an expression of universal cause and effect: one reaps what one sows, the quality of one’s actions is returned in kind, there is virtue for virtue and evil for evil (Holt, 2011; Chen et al., 2018). Chinese DCWs utilize *bao* in the distinct context of the nursing home to navigate demanding work conditions, such as understaffing, and the concerning prospects of their own constrained elder care resources. In the Chinese cultural context, *bao* depicts the rules of interaction-based trust development (Luo, 2005). Two features contained in *bao* distinguish it from the Western norm of reciprocity: one feature is that *bao* is a long-term oriented exchange that discourages instant bargaining or payback; the other is that a *bao*-induced relationship creates affection (Liang et al., 2018). According to this cultural logic, DCWs in this study hope that their current care for older adults will be returned when their own care needs arise in the future, instead of expecting immediate pay raises from their work. Though this helps to imbue their mundane tasks of care with a moral dimension and facilitates a caring relationship with older adults, what makes Chinese DCWs’ concerns for their future unique is also, in part, a result of the unintended consequence of China’s already abolished one child policy, which has created a sudden and surging demand for a formal care workforce (Alpermann & Zhan, 2019), ready or not.

### Practical Implications

The nonreciprocal elements of care work identified in this study might perpetuate devaluations of direct care if left unattended. Therefore, recognizing and supporting the emotional demands of care work is crucial to strengthening the workforce (Franzosa et al., 2019). However, targeted emotional support for DCWs to manage emotional distress is inadequate. While standardization and professionalization of care services ensure unified practices of care, the emotional aspects of care work, such as putting on a *genuine* smile, are hard to mandate. Several implications can be drawn from this article.

First, training and capacity building programs which improve DCWs’ professional skills need to be developed and put in place. Considering DCWs’ level of education, these programs should be more practice-driven than theoretical. Professional skills and identity can serve as starting points from which DCWs can address devaluations of care work, enhance their self-esteem, and reduce emotional burnout. Second, social service work can be developed to aid DCWs in navigating emotional stress and burdens at the workplace and achieve a work-life balance. Such initiatives find resonance in the culture change movement in U.S. nursing homes. Its philosophy embraces person-centered concepts, while also supporting the improvement of work conditions for staff (Koren, 2010). This way, the importance of emotions in DCWs’ labor processes could be formally acknowledged and intervention programs could be developed accordingly which address the experiential needs of DCWs. Third, social protection for DCWs should be enhanced by relevant government agencies through a formalization of the working status of DCWs, including adequate welfare entitlements to address their concerns for their own elder care. Existing public subsidies and other benefits for nursing home operators should be based on evaluations of services targeted for not only older adults, but also frontline staffers, including DCWs. These would help to mitigate the nonreciprocal elements of care work.

### Limitations

This study has several limitations. First, the source of data was from one government-sponsored nursing home. Therefore, the findings from this study and the implications it generates should be treated with caution and interpreted within the distinct nursing home environment. Presumably, DCWs in private nursing homes might experience emotional labor differently due to the market logic which often prioritizes efficiency over time-consuming emotional care. Future research could explore and compare how emotional labor is similarly or differently experienced in nursing homes of various ownership configurations. Second, this study did not capture the experience of DCWs who have already left the sector. Arguably, DCWs who remain in elder care might more readily identify with their caregiving role and accept the work conditions as an extension of their caring personalities. Thus, capturing the voice of DCWs who voted with their feet in future research could provide a more comprehensive picture of diverse caregiving experiences and inform LTC policymaking. Additionally, given the imbalanced development of care service in urban and in rural China, future research could explore how features of emotional labor identified in this study (e.g. caring, professional, reciprocal) manifest in rural nursing homes where care resources are scant. In spite of these limitations, as an exploratory effort, this study contributes and enriches understanding of the daily care experiences of nursing home DCWs in a rapidly aging China through the theoretical lens of emotional labor. As China is where the largest number of the world’s silver population reside, the issue of the care workforce needs special attention to ensure the welfare of both older adults and those who care for them as Chinese people live longer and healthier lives.

## Conclusion

This article delves into the manifestation of emotional labor among Chinese DCWs, a group of service workers whose labor is devalued and stigmatized, and an underexplored topic in China’s aging studies. Although results from this study might not apply to nursing homes of different types or in other regions in China, the three identified themes in this article suggest the implicit meanings and often-conflicting nature of nursing home care work; a still less respected but fast-growing profession. The emotional labor with which DCWs enact the process of managing their emotions and inner feelings in line with the interest of older adults is simultaneously rewarding and challenging. Additional to insufficient recognition of their work, the looming concern behind their self-perceived caring and professional identities is DCWs’ uncertainty over their own elder care prospects. As a coping strategy, DCWs evoke the notion of *bao* to assuage the inadequacy of care resources as family caregiving capacity diminishes and care cost increases. This enables DCWs to temporarily remain hopeful and focus their energy on caring for older adults, which is in line with the interest of their employer. Importantly, this study has demonstrated, through detailed accounts of the care experiences of DCWs in a Chinese nursing home, that the emotional burden derived from structural deficiencies in the care system (e.g. heavy workloads and understaffing) should not be reductively and expediently attributed to DCWs’ incapacities and lack of professionalism in navigating the complex and unpredictable care needs of older adults.

## Footnotes

1 1 All names are pseudonyms.
